# ERAP1-ERAP2 dimers trim MHC I-bound precursor peptides; implications for understanding peptide editing

**DOI:** 10.1038/srep28902

**Published:** 2016-08-12

**Authors:** Hanna Chen, Lenong Li, Mirjana Weimershaus, Irini Evnouchidou, Peter van Endert, Marlene Bouvier

**Affiliations:** 1 Department of Microbiology and Immunology, University of Illinois at Chicago, College of Medicine, Chicago, IL 60612 USA; 2 INSERM, Unité 1151; CNRS, Unité 8352; Université Paris Descartes, 75015 Paris, France

## Abstract

The processing of MHC class I antigenic precursor peptides by the endoplasmic reticulum aminopeptidase 1 (ERAP1) and ERAP2 is an important event in the cell biology of antigen presentation. To date, the molecular context by which the ERAP enzymes trim precursor peptides, and how ERAPs shape peptide repertoires, remain open questions. Using ERAP1 and ERAP2 heterodimers (ERAP1/2), and N-terminally extended model and natural peptides in their free and HLA-B*0801-bound forms, we characterized the mode of action of ERAPs. We provide evidence that ERAP1/2 can trim MHC I-bound precursor peptides to their correct and final lengths, albeit more slowly than the corresponding free precursors. Trimming of MHC I-bound precursors by ERAP1/2 increases the conformational stability of MHC I/peptide complexes. From the data, we propose a molecular mechanistic model of ERAP1/2 as peptide editors. Overall, our study provides new findings on a significant issue of the ERAP-mediated processing pathway of MHC class I antigens.

The elimination of infected cells by cytotoxic T lymphocytes (CTLs) is critically dependent on the cell-surface presentation of antigenic peptides by MHC class I molecules. These molecules are assembled and loaded with peptides within the endoplasmic reticulum (ER). The quality of these events is controlled by a protein machinery made up of calreticulin, the thiol oxidoreductase ERp57, tapasin, and the peptide transporter associated with antigen processing (TAP)^1^,^2^; this machinery is referred to as the peptide-loading complex (PLC). We, and others, have shown that tapasin stabilizes immature MHC I molecules^3^,^4^,^5^,^6^,^7^,^8^, enabling it to exert a critical quality control editing function towards bound candidate peptides^4^.

Most antigenic peptides are generated first in the cytosol as precursors by the proteasome^2^,^9^. These precursors have the correct C-termini of the final antigenic peptides, but the N-termini carry one or several extra residues. In humans, these N-terminal residue extensions are trimmed in the ER by the closely related enzymes ERAP1 (equivalent to mouse ERAP) and ERAP2^10^,^11^,^12^. It was shown that mice lacking ERAP have a significantly altered MHC I-restricted peptide repertoire relative to wild-type mice^13^,^14^,^15^,^16^,^17^,^18^, suggesting a role for ERAPs in peptide editing quality control. To date, the molecular context by which ERAP1 and ERAP2 trim precursors to the final peptides, and how the enzymes influence the development of antigen repertoires, remain open and debated questions.

It was suggested that mouse ERAP synergizes with MHC I molecules for trimming precursor peptides to their final lengths^19^. The possibility that MHC I molecules themselves play a substantial role in the intracellular generation of peptides was in fact proposed even before the initial discovery of aminopeptidases^20^. Others have suggested instead that ERAP1 relies on an intrinsic ruler mechanism for trimming free precursors^21^. The x-ray crystal structures of ERAP1 (without a bound peptide substrate) and ERAP2 (with and without bound peptide substrates) have described enzyme conformations that appear to be more suited for trimming free than MHC I-bound peptides^22^,^23^,^24^,^25^,^26^. Nevertheless, several arguments suggest that trimming of MHC I-bound peptides by ERAP1 and ERAP2 is a plausible mode of action. First, the finding that mouse ERAP influences peptide repertoires^13^,^14^,^15^,^16^,^17^,^18^ can be more easily comprehended mechanistically if there is a direct molecular cross-talk between ERAP and MHC I/precursor complexes. Second, it was shown in ERAP1-deficient cells that a N-terminally extended peptide that is disulfide-linked as a single-chain MHC I trimer (comprising heavy chain (HC) and β_2_m) stimulated T-cell activities only upon co-expression of ERAP1^27^. Third, ERAP1 and ERAP2 polymorphisms are associated with the genetic risk of contracting autoimmune diseases such as ankylosing spondylitis and birdshot retinopathy^28^. In formulating explanations for these genetic linkages, the questions of *if/how* ERAPs interact with disease-associated MHC I molecules *have to* be raised.

Here, we developed an *in vitro* system composed of ERAP1/2^29^, MHC I molecules, and N-terminally elongated peptides, and demonstrate that ERAP1/2 can trim MHC I-bound precursors, in addition to free precursors. We provide a molecular mechanistic understanding of how ERAP-mediated processing of MHC I-bound precursors can exert an editing quality control function to generate stable MHC I/peptide complexes.

## Results

### Precursor peptides

We designed model precursor peptides based on the HLA-B*0801-restricted ELRSRYWAI 9mer from the nucleoprotein of influenza virus^30^. A Glu-to-Ala mutation was introduced at P1 because of the low specificity that ERAPs have for glutamic acid^31^. The ALRSRYWAI peptide was N-terminally extended with arginine, favorably processed by ERAPs, and alanine thereby generating (RA)_n_ALRSRYWAI (n = 2 and 3). Other similar model precursors were based on the HLA-B*0801-restricted GGKKKYKL 8mer, derived from the HIV-1 Gag protein^32^, in which Gly-to-Ala mutations were introduced at P1 and P2, because of the low specificity of ERAPs for glycine^31^, yielding (RA)_n_AAKKKYKL (n = 2 and 3).

### Binding of precursor peptides onto MHC I molecules

**Figure 1:**
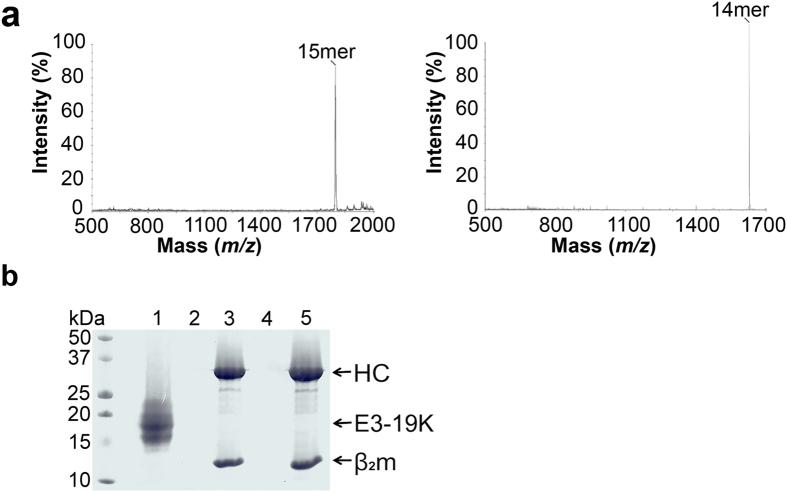
Characterization of HLA-B*0801/precursor complexes. (**a**) MALDI-TOF MS analysis of HLA-B*0801/(RA)_3_ALRSRYWAI and HLA-B*0801/(RA)_3_AAKKKYKL complexes shows peaks corresponding to (RA)_3_ALRSRYWAI 15mer (*m/z* = 1817) and (RA)_3_AAKKKYKL 14mer (*m/z* = 1631) ligands. (**b**) Ni-NTA agarose beads were used in the capture of Ad4 E3-19K(His)_6_ (lane 1), HLA-B*0801/ALRSRYWAI (lane 2), HLA-B*0801/(His)_6_ALRSRYWAI (lane 3), HLA-B*0801/AAKKKYKL (lane 4), and HLA-B*0801/(His)_6_AAKKKYKL (lane 5). The supernatants of the pelleted, washed, and boiled beads were loaded on SDS-PAGE gel (15%).

Precursor peptides were incubated with peptide-deficient HLA-B*0801 molecules as described in Methods. The controls consisted of the corresponding free peptides incubated alone. The sample and control mixtures were washed extensively in mini-spin columns to remove free peptides, after which the retentate fractions containing HLA-B*0801/peptide complexes were analyzed by mass spectrometry (MS) (see Methods). Typical analyses for HLA-B*0801/(RA)_3_ALRSRYWAI and HLA-B*0801/(RA)_3_AAKKKYKL show peaks corresponding to the 15mer and 14mer precursor ligands (Fig. 1a). It is important to note that free peptides were efficiently removed in this assay as no signal peaks for peptides were found from analyses of the control retentates. Moreover, HLA-B*0801/precursor complexes consistently migrated as single compact bands on native PAGE gel, in contrast to the protein smears normally observed for peptide-deficient MHC I molecules alone. This suggests that the precursors provide some amount of stability to HLA-B*0801 molecules.

### Precursor Peptides Extend out of the MHC I Groove

To ascertain that the N-terminus of the precursor peptides protrude out of the MHC I groove, we tested if HLA-B*0801/(His)_6_ALRSRYWAI and HLA-B*0801/(His)_6_AAKKKYKL can be captured by Ni-NTA beads through their poly-His peptide tags. The controls consisted of HLA-B*0801/ALRSRYWAI, HLA-B*0801/AAKKKYKL, and Adenovirus serotype 4 (Ad4) E3-19 K(His)_6_. The samples and controls were incubated with Ni-NTA beads, followed by analysis of the washed beads on SDS-PAGE gel (Fig. 1b). Results show that HLA-B*0801/(His)_6_ALRSRYWAI (lane 3) and HLA-B*0801/(His)_6_AAKKKYKL (lane 5) were captured by the Ni-NTA resin as evidenced by the HC and β_2_m bands. The negative controls HLA-B*0801/ALRSRYWAI (lane 2) and HLA-B*0801/AAKKKYKL (lane 4) could not be captured, whereas the positive control Ad4 E3-19 K(His)_6_ (lane 1) was revealed as several bands corresponding to glycosylated Ad4 E3-19 K(His)_6_. Together these results demonstrate that the N-terminus of (His)_6_ALRSRYWAI and (His)_6_AAKKKYKL is solvent-accessible in their HLA-B8-bound forms.

### ERAP1/2 trims MHC I-bound (RA)_n_ALRSRYWAI precursors

**Figure 2:**
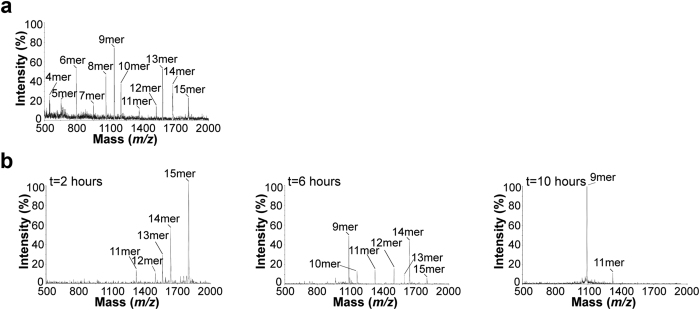
N-terminal trimming of free and HLA-B*0801-bound (RA)_3_ALRSRYWAI by ERAP1/2. (**a**) Free (RA)_3_ALRSRYWAI 15mer was incubated with ERAP1/2 at 37 °C. An aliquot was taken from the mixture after 1 hour 20 minutes and analyzed by MALDI-TOF MS. (**b**) HLA-B*0801-bound (RA)_3_ALRSRYWAI 15mer was incubated with ERAP1/2 at 37 °C. Aliquots were taken after 2, 6, and 10 hours and analyzed by MALDI-TOF MS. Initially, the enzyme:substrate ratio was as identical as possible in (**a**,**b**), but additional ERAP1/2 was added in (**b**) after 2 and 6 hours. The starting precursor peptides and their fragments are identified. See also Supplementary Fig. 1 for (RA)_2_ALRSRYWAI 13mer, and Supplementary Fig. 2 and 3 for (RA)_n_AAKKKYKL series.

We first monitored the trimming of free (RA)_3_ALRSRYWAI 15mer precursor by ERAP1/2 (Fig. 2a). Results show that trimming of the 15mer produced fragments as short as 4mer. It is noteworthy that the ALRSRYWAI 9mer is trimmed to 8mer by ERAP1/2. Similar experiments using the (RA)_2_ALRSRYWAI 13mer yielded identical fragments (Supplementary Fig. 1a).

Next, the ability of ERAP1/2 to trim HLA-B*0801-bound (RA)_3_ALRSRYWAI was examined under similar conditions (Fig. 2b). Results obtained after 2 hours show that hydrolysis of the starting HLA-B*0801-bound 15mer yielded 14mer as the major fragment. After 6 hours, and with additional ERAP1/2, the shortest fragment detected was the ALRSRYWAI 9mer. Finally, after 10 hours, the 9mer was the major product with only traces of the 11mer. These results are in marked contrast to those obtained for trimming of free (RA)_3_ALRSRYWAI, which produced fragments shorter than 9mer (Fig. 2a). It is also noteworthy that the 9mer fragment was detected at a much later time in this mixture relative to digestion of free (RA)_3_ALRSRYWAI (compare Fig. 2a-2b, respectively). Similar results were obtained for HLA-B*0801-bound (RA)_2_ALRSRYWAI 13mer (Supplementary Fig. 1b), where the endpoint of hydrolysis after 10 hours was the 9mer, and only the 9mer. Again, the 9mer was generated significantly more slowly from MHC I-associated than from free substrates. Importantly, a control in which HLA-B*0801-bound (RA)_2_ALRSRYWAI was incubated in the absence of ERAP1/2 showed no evidence that (RA)_2_ALRSRYWAI is trimmed spontaneously (Supplementary Fig. 1c), confirming that our results are specifically due to the action of ERAP1/2. Overall, these experiments provide evidence that ERAPs removed completely the (RA)_n_ extension of HLA-B*0801-bound (RA)_n_ALRSRYWAI, generating MHC I-associated ALRSRYWAI.

### Stabilities of MHC I/precursor complexes

We next assessed the stabilities of HLA-B*0801/precursor complexes under the conditions of our trimming assay (see Methods). Using equal amounts of HLA-B*0801/ALRSRYWAI (control) and HLA-B*0801/(RA)_3_ALRSRYWAI, we determined by native PAGE gel analysis that 86% and 74%, respectively, of complexes could be quantitatively recovered after 10 hours incubation with ERAP1/2 at 37 °C. Therefore, HLA-B*0801/intermediate complexes are overall quite stable, with a loss of only ~10% greater than that of the control complex.

It is possible that the loss of HLA-B^*^0801/precursor complexes measured above is due to precursor release from the groove. To test if peptide-deficient HLA-B*0801 molecules that would then be generated can re-bind peptides, we incubated HLA-B*0801/(RA)_2_ALRSRYWAI (Tm = 43.8 °C) with an excess of free AAKKKYKL 8mer for 10 hours at 37 °C, in the absence of ERAP1/2, under the conditions of our trimming assay (see Methods) – importantly, note that AAKKKYKL forms very stable complexes with HLA-B*0801, Tm = 66.7 °C. An analysis of the retentate fraction corresponding to HLA-B*0801/peptide complexes by MS (Supplementary Fig. 1d), after washing away free peptides, show a strong peak for (RA)_2_ALRSRYWAI 13mer ligand but no peak for AAKKKYKL 8mer (*m/z* = 949). Therefore, we conclude that if the loss of HLA-B*0801/precursor complexes over time arises from precursor release, the peptide-deficient HLA-B*0801 molecules generated *in situ* cannot re-bind peptides, most likely because these molecules are unstable at 37 °C and degrade rapidly as we showed previously^33^ (see also results below on disulfide-linked peptide). It is also possible that the lost of HLA-B*0801/precursor complexes over time arises via other mechanisms such as the overall denaturation of complexes.

### ERAP1/2 trims MHC I-bound (RA)_n_AAKKKYKL precursors

To confirm the results obtained with N-terminally extended ALRSRYWAI 9mer, similar experiments were carried out with a second N-terminally extended peptide based on the AAKKKYKL 8mer. Trimming of free (RA)_3_AAKKKYKL 14mer produced fragments as short as 4mer (Supplementary Fig. 2a). A similar experiment using the (RA)_2_AAKKKYKL 12mer yielded nearly identical fragments (Supplementary Fig. 3a).

The HLA-B*0801-bound (RA)_3_AAKKKYKL 14mer was then incubated with ERAP1/2 (Supplementary Fig. 2b). After 2 hours, the starting 14mer was trimmed to a series of products, with the 13mer being the dominant fragment. After 6 hours, the 9mer became dominant. Finally, after 10 hours, the AAKKKYKL 8mer peptide was the only product detected. These results reproduced our observations with MHC I-bound (RA)_n_ALRSRYWAI in that trimming of the precursor proceeded more slowly but stopped at the optimal MHC I-adapted peptide length, while the free precursor was degraded faster and to shorter products (compare Supplementary Fig. 2a and 2b, respectively). Similar results were obtained for HLA-B*0801-bound (RA)_2_AAKKKYKL 12mer (Supplementary Fig. 3b). Overall, our findings on trimming of HLA-B*0801-bound precursors by ERAP1/2 are duplicated with both the (RA)_n_ALRSRYWAI and (RA)_n_AAKKKYKL series.

### Characterization of ERAP active species

We next asked whether trimming of MHC I-bound precursors could be attributed to one of the two enzymes in the ERAP1 and ERAP2 heterodimers. To address this question, we used ERAP1/2 in which ERAP1 was inactivated by the single-point mutation E354A^29^. ERAP1/2 containing inactive ERAP1 trimmed either forms of (RA)_3_ALRSRYWAI significantly less efficiently (Supplementary Fig. 4); no fragments smaller than 9mer were generated for free (RA)_3_ALRSRYWAI (Supplementary Fig. 4a), or smaller than 12mer for HLA-B*0801-bound (RA)_3_ALRSRYWAI (Supplementary Fig. 4b, 10 hours). The addition of more ERAP1/2 beads did not alter these MS profiles. Thus, active ERAP2 alone displayed poor trimming activity towards both free and HLA-B*0801-bound precursors.

### Jun linkage

To rule out any potential effects of the Jun modification on ERAP1 activity, we compared the trimming of free (RA)_3_ALRSRYWAI using ERAP1 beads that were generated without and with a Jun linkage (Supplementary Fig. 4c). Our results show that there are essentially no differences in the kinetics and extent of trimming of free (RA)_3_ALRSRYWAI between the two types of beads. A similar analysis using HLA-B*0801-bound (RA)_3_ALRSRYWAI confirmed this conclusion. These results validate our *in vitro* system based on ERAP beads.

### ERAP1/2 trims disulfide-linked HLA-B*0801E76C/(RA)_3_AAKKKYCL

**Figure 3:**
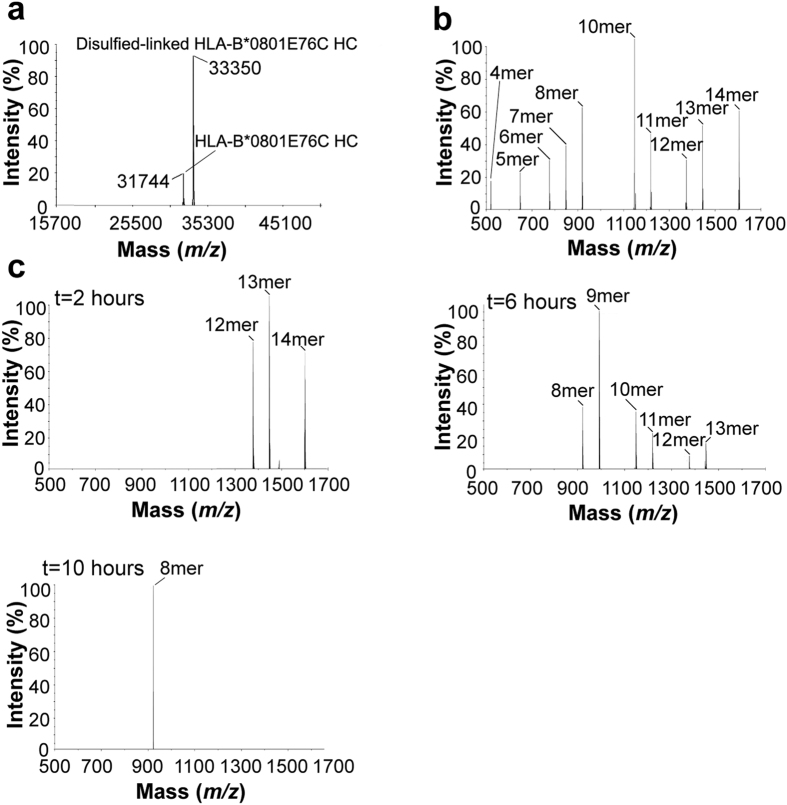
N-terminal trimming of free (RA)_3_AAKKKYCL and disulfide-linked HLA-B*0801E76C/(RA)_3_AAKKKYCL by ERAP1/2. (**a**) ESI LC MS analysis of HLA-B*0801E76C/(RA)_3_AAKKKYCL. The major peak corresponds to disulfide-linked HLA-B*0801E76C HC (*m/z* = 33350) and the minor peak corresponds to HLA-B*0801E76C HC (*m/z* = 31744). (**b**) Free (RA)_3_AAKKKYCL was incubated with ERAP1/2 at 37 °C. An aliquot was taken from the mixture after 1 hour 20 minutes and analyzed by ESI LC MS. (**c**) Disulfide-linked HLA-B*0801E76C/(RA)_3_AAKKKYCL was incubated with ERAP1/2 at 37 °C. Aliquots were taken from the mixture after 2, 6, and 10 hours and analyzed by ESI LC MS. Initially, the enzyme:substrate ratio was as identical as possible in (**b**,**c**), but additional ERAP1/2 was added in (**c**) after 2 and 6 hours. The starting precursor peptides and their fragments are identified.

To provide further evidence that ERAP1/2 trims MHC I-bound precursor substrates, we generated the disulfide-linked HLA-B*0801E76C/(RA)_3_AAKKKYCL complex, in which (RA)_3_AAKKKYCL is covalently trapped within the groove thereby preventing its release as a free peptide. (RA)_3_AAKKKYCL was disulfide bonded via a C-terminal residue (P7) to ensure that N-terminal peptide residues are accessible to ERAP1/2. An analysis of HLA-B*0801E76C/(RA)_3_AAKKKYCL by MS (Fig. 3a) show that disulfide bond formation between (RA)_3_AAKKKYCL and HLA-B*0801E76C proceeded efficiently as evidenced by a major peak *(m/z* = 33350) corresponding to disulfide-linked HLA-B*0801E76C HC; the minor peak (*m/z* = 31744) corresponds to HLA-B*0801E76C HC and likely arises from peptide-deficient HLA-B*0801E76C molecules that are not peptide-receptive, especially because a peak corresponding to free (RA)_3_AAKKKYCL could not be detected in the spectrum. Importantly, we tested that the disulfide-linkage in HLA-B*0801E76C/(RA)_3_AAKKKYCL is stable under the conditions of our trimming assay after incubation of the complex for 10 hours at 37 °C (see Methods); the mixture was spun-down in a mini-spin column and an analysis of the concentrated flow-through fraction by MS showed no evidence of (RA)_3_AAKKKYCL in solution.

Notably, trimming of free (RA)_3_AAKKKYCL by ERAP1/2 generated fragments as short as 4mer (Fig. 3b), while trimming of disulfide-linked (RA)_3_AAKKKYCL generated AAKKKYCL 8mer as the exclusive final product (Fig. 3c, 10 hours). Therefore, results from the disulfide-linked (RA)_3_AAKKKYCL provide further evidence that ERAP1/2 trims MHC I-bound peptides, as (RA)_3_AAKKKYCL could not be released as a free peptide during the trimming assay. Taken together, our combined results support the idea that precursor peptides bound to HLA-B*0801 molecules are trimmed by ERAPs “on MHC I”, and are inconsistent with a model in which the precursors are released from HLA-B*0801, trimmed in solution by ERAPs, followed by their re-binding onto peptide-deficient molecules formed *in situ*.

### Stability of HLA-B*0801/(RA)_3_ **ALRSRYWAI** under the action of ERAP1/2

**Figure 4:**
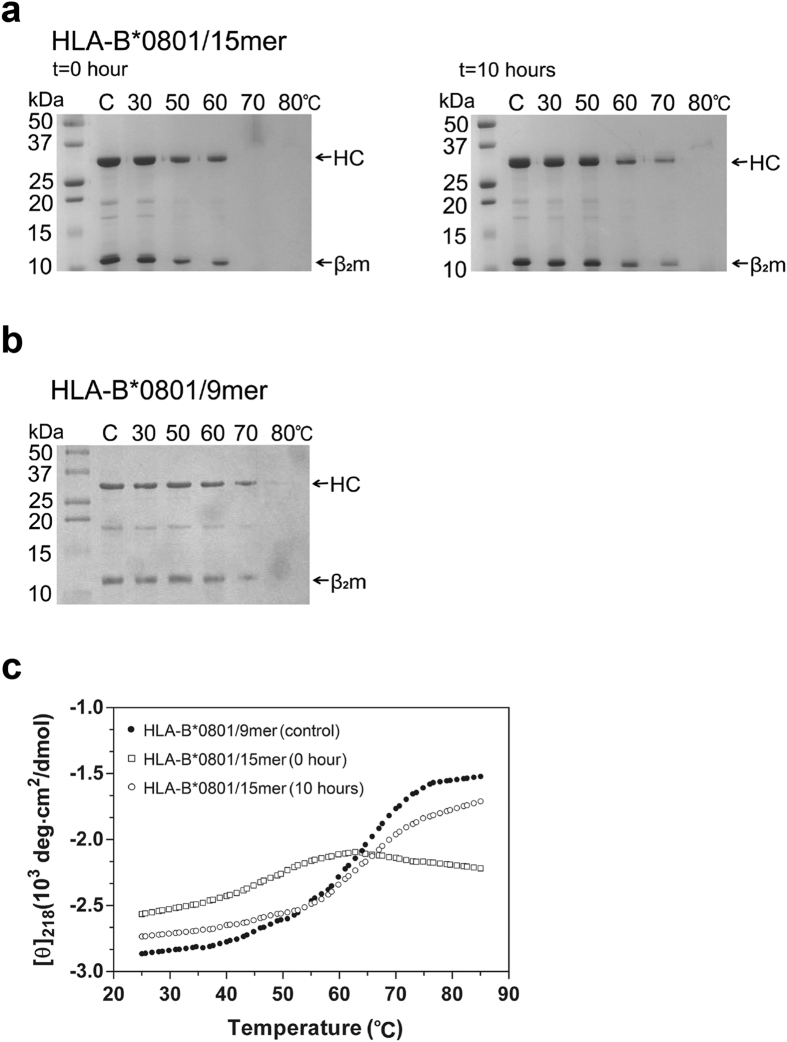
Thermal stability of HLA-B*0801/(RA)_3_ALRSRYWAI before and after incubation with ERAP1/2. (**a**) Thermolytic digests of HLA-B*0801**/**(RA)_3_ALRSRYWAI were carried out at the indicated temperatures before (t = 0 hour) and after (t = 10 hours, generated HLA-B*0801**/**ALRSRYWAI) incubation with ERAP1/2. The enzyme:substrate ratio was 1:300 (W/W) and digest times were 15 minutes. Samples were analyzed on SDS-PAGE gel (15%). The control lane, labeled C, represents protein without added thermolysin and loaded at the same concentration as in the other lanes. (**b**) Thermolytic digests of HLA-B*0801/ALRSRYWAI generated from direct peptide binding. Conditions are as given in (**a**). (**c**) Circular dichroism thermal denaturation curves for HLA-B*0801/peptide complexes described in (**a**,**b**). See also Supplementary Fig. 5.

We next used the enzyme thermolysin to examine how the trimming of HLA-B*0801-bound precursors by ERAP1/2 affects the stability of HLA-B*0801/peptide complexes. We carried out digests, at different temperatures, of HLA-B*0801/(RA)_3_ALRSRYWAI complexes before (t = 0 hour) and after (t = 10 hours) incubation with ERAP1/2, followed by analysis of the mixtures by SDS-PAGE (Fig. 4a). A control consisting of HLA-B*0801/ALRSRYWAI, generated from binding ALRSRYWAI onto HLA-B*0801, was similarly characterized (Fig. 4b). Results show that the starting HLA-B*0801/(RA)_3_ALRSRYWAI complex (Fig. 4a, t = 0 hour) is stable up to about 50 °C–60 °C as evidenced by a strong HC band. However, it is denatured at 70 °C as seen by the disappearance of HC. In contrast, analysis of HLA-B*0801/(RA)_3_ALRSRYWAI after incubation with ERAP1/2 for 10 hours (Fig. 4a), at which time trimming generated HLA-B*0801/ALRSRYWAI (see Fig. 2b), shows that the complex is denatured at 80 °C as seen by the disappearance of the HC band. Thus, trimming of HLA-B*0801/(RA)_3_ALRSRYWAI by ERAP1/2 generated a more heat-resistant complex, i.e., HLA-B*0801/ALRSRYWAI. Importantly, complexes generated either from ERAP1/2 trimming of HLA-B*0801/(RA)_3_ALRSRYWAI (10 hours) or direct loading of ALRSRYWAI onto HLA-B*0801 molecules show similar band patterns (compare Fig. 4a (10 hours) and 4b, respectively) consistent with the comparable thermal stability of these complexes, as expected.

We also used circular dichroism (CD) to monitor the thermal stability of HLA-B*0801/(RA)_3_ALRSRYWAI before (t = 0 hour) and after (t = 10 hours) incubation with ERAP1/2 (Fig. 4c). Analysis of thermal denaturation curves shows that the mid-point temperature (Tm) of HLA-B*0801/15mer increased from 48.0 °C (open square) to 62.6 °C (open circle) upon incubation with ERAP1/2. A control consisting of HLA-B*0801/ALRSRYWAI, generated from binding ALRSRYWAI onto HLA-B*0801, displayed a Tm of 63.2 °C (closed circle), nearly identical to that of HLA-B*0801/(RA)_3_ALRSRYWAI generated after 10 hours incubation with ERAP1/2. In conclusion, thermolysin and CD experiments showed that trimming of (RA)_3_ residues from HLA-B*0801-bound (RA)_3_ALRSRYWAI by ERAP1/2 enhances the stability of HLA-B*0801 molecules.

### Stability of HLA-B*0801/LAKLRNKLVI under the action of ERAP1/2

To further confirm that trimming of HLA-B*0801-bound precursor peptides by ERAP1/2 enhances the stability of HLA-B*0801 molecules, we used two peptides that differ by only a single N-terminal residue; LAKLRNKLVI 10mer and AKLRNKLVI 9mer (Supplementary Fig. 5). After 4 hours, HLA-B*0801-bound LAKLRNKLVI 10mer was trimmed to AKLRNKLVI 9mer, and only 9mer (Supplementary Fig. 5a). No further cleavage of HLA-B*0801/AKLRNKLVI was observed after longer incubation times and with additional ERAP1/2. This is entirely consistent with results for HLA-B*0801/AKLRNKLVI complexes which we showed are insensitive to ERAP1/2 trimming over a period of 4 hours (Supplementary Fig. 5b). Here again, thermal denaturation analyses by CD showed that HLA-B*0801/LAKLRNKLVI is less stable than HLA-B*0801/AKLRNKLVI; compare Tm = 52.6 °C with Tm = 62.8 °C, respectively. Overall, these experiments provide further evidence that trimming of HLA-B*0801-bound precursors by ERAP1/2 enhances the stability of HLA-B*0801/peptide complexes.

### ERAP1/2 trimming of long natural peptides to epitopes

**Figure 5:**
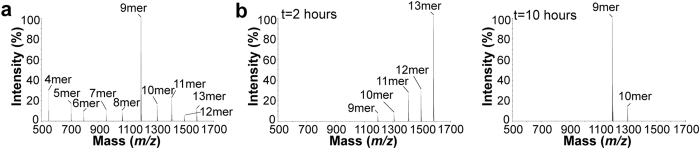
N-terminal trimming of a long natural peptide SSTLELRSRYWAI by ERAP1/2. (**a**) Free SSTLELRSRYWAI 13mer was incubated with ERAP1/2 at 37 °C. An aliquot was taken from the mixture after 2 hours and analyzed by ESI LC MS. (**b**) HLA-B*0801-bound SSTLELRSRYWAI 13mer was incubated with ERAP1/2 at 37 °C. Aliquots were taken after 2 and 10 hours and analyzed by ESI LC MS. Initially, the enzyme:substrate ratio was as identical as possible in (**a**,**b**), but additional ERAP1/2 was added in (**b**) after 2 and 6 hours. The starting precursor peptides and their fragments are identified.

We examined the role of ERAP1/2 in trimming long natural peptides to immunodominant epitopes. For this, we used the SSTLELRSRYWAI 13mer from the nucleoprotein of influenza virus that contains the HLA-B*0801-restricted ELRSRYWAI 9mer epitope^30^; SSTLELRSRYWAI is a hypothetical cytosolic precursor that depends on TAP for transport into the ER^34^. The 13mer was incubated with ERAP1/2 as a free and HLA-B*0801-bound peptide (Fig. 5). Results show that while free SSTLELRSRYWAI was trimmed to fragments as short as 4mer, with the 9mer being dominant, after 2 hours (Fig. 5a), trimming of HLA-B*0801-bound SSTLELRSRYWAI produced no fragments smaller than 9mer after 10 hours (Fig. 5b). Again, the 9mer was generated significantly more slowly from the HLA-B*0801-associated form relative to its free form (compare Fig. 5a-5b, respectively). Together, these results show that the ELRSRYWAI epitope was produced from trimming a long natural peptide in both its free and MHC I-bound forms, with trimming of the free form being a faster process kinetically.

**Figure 6:**
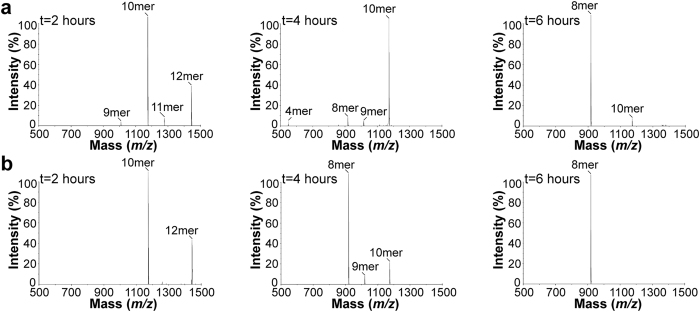
N-terminal trimming of a long natural peptide RLRPGGKKKYKL by ERAP1/2. (**a**) Free RLRPGGKKKYKL 12mer was incubated with ERAP1/2 at 37 °C. An aliquot was taken from the mixture after 2, 4, and 6 hours and analyzed by ESI LC MS. (**b**) HLA-B*0801-bound RLRPGGKKKYKL 12mer was incubated with ERAP1/2 at 37 °C. Aliquots were taken after 2, 4, and 6 hours and analyzed by ESI LC MS. The enzyme:substrate ratio was as identical as possible in (**a**,**b**), and additional ERAP1/2 was added in (**b**) after 2, 4, and 6 hours. The starting precursor peptides and their fragments are identified.

We also tested the long natural peptide RLRPGGKKKYKL, a 12mer derived from the HIV-1 Gag protein^32^. Notably, RLRPGGKKKYKL contains the HLA-B*0801-restricted GGKKKYKL 8mer epitope^32^ and HLA-B*0702-restricted RPGGKKKYKL 10mer epitope^35^. First, the RLRPGGKKKYKL 12mer was incubated with ERAP1/2 as a free and HLA-B*0801-bound substrate (Fig. 6). Trimming of free RLRPGGKKKYKL for 4 hours generated the 10mer as the dominant fragment (Fig. 6a). The 10mer was trimmed to the 8mer epitope after 6 hours. The composition of the mixture did not change after 6 hours incubation, i.e. the endpoint was the GGKKKYKL 8mer epitope. In contrast, incubation of HLA-B*0801/RLRPGGKKKYKL with ERAP1/2 (Fig. 6b) showed that the HLA-B*0801-bound GGKKKYKL 8mer was already dominant after 4 hours and the exclusively generated product after 6 hours. Thus, in marked contrast to our previous examples, the 8mer was generated more rapidly when RLRPGGKKKYKL was trimmed in its HLA-B*0801-bound form. Importantly, thermal denaturation experiments showed that RPGGKKKYKL 10mer forms more unstable complexes with HLA-B*0801 relative to GGKKKYKL 8mer; compare Tm = 46.6 °C versus Tm = 62.0 °C respectively. In separate experiments, we found that incubation of HLA-B*0801/RPGGKKKYKL with ERAP1/2 also yielded HLA-B*0801/GGKKKYKL as the final product. Here again, the kinetics for generating the 8mer was faster when RPGGKKKYKL was trimmed in its HLA-B*0801-bound form. Taken together, the HLA-B*0801-restricted GGKKKYKL 8mer epitope was generated from trimming a long natural peptide in both its free and MHC I-bound forms, with trimming being faster kinetically for the MHC I-associated form.

Finally, only RPGGKKKYKL 10mer, and not LRPGGKKKYKL 11mer and RLRPGGKKKYKL 12mer, could be loaded onto peptide-deficient HLA-B*0702 molecules. The resulting HLA-B*0702/10mer complex was however insensitive to trimming by ERAP1/2, even after long incubation times and with additional ERAP1/2. This shows that, in contrast to HLA-B*0801-restricted precursors, the HLA-B*0702-restricted RPGGKKKYKL 10mer epitope could be formed only from trimming of a free natural precursor.

Taken together, the above results provide some insights into the relative roles that free and MHC I-bound precursor trimming by ERAP1/2 play in the actual generation of MHC I peptide repertoires *in vivo*.

### Model of a MHC I-bound precursor peptide

**Figure 7:**
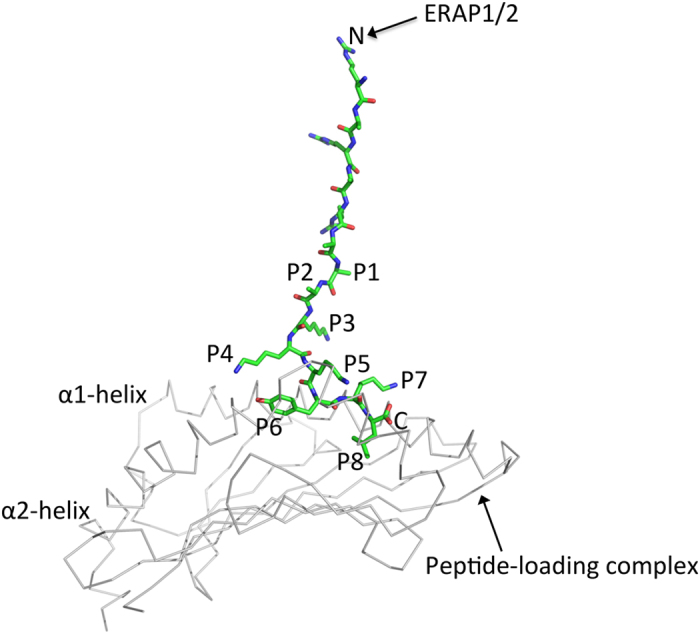
A model showing how (RA)_3_AAKKKYKL 14mer can bind within the groove of HLA-B*0801. The model is based on the x-ray structure of HLA-B*0801/GRKKKYKL (PDB code 1AGB), and built using AAKKKYKL 8mer. We suggest that the peptide-loading complex provides stabilizing energy to the conformationally immature HLA-B*0801/(RA)_3_AAKKKYKL as (RA)_3_AAKKKYKL undergoes N-terminal trimming “on MHC I” by ERAP1/2 to the final AAKKKYKL 8mer (see Discussion). The model was built and minimized using the UCSF Chimera package^43^. Hydrogen atoms were added to the model, and the AMBER 14SB force field, with atom and bond parameters from the AM1-BCC charge model, was used for calculations. The α1- and α2-helices, and N- and C-termini are indicated.

Based on our data, we propose a model of how a precursor such as (RA)_3_AAKKKYKL could possibly bind within the groove of HLA-B*0801 (Fig. 7). The model is based on the crystal structure of HLA-B*0801/GGKKKYKL (PDB code 1AGB) and shows five N-terminal residues AAKKK (P1-P5), carrying the (RA)_3_ extension, protruding out of the groove and three C-terminal residues YKL (P6-P8) bound within the groove. It is entirely conceivable that 1 or 2 more C-terminal residues are bound within the groove, and that the stretch of N-terminal residues in the solvent space undergoes conformational changes. This model of a long precursor binding to MHC I incorporates several important physiological points: (1) the low stabilities of MHC I/precursor complexes (see Fig. 4), reflecting largely the lack of critical hydrogen bonds between the peptide amino terminus and MHC I residues at the N-terminal end of the groove^36^; (2) the critical role that C-terminal peptide residues play in anchoring peptides within the F pocket^37^,^38^; (3) a stabilizing role for PLC proteins at the C-terminus of MHC I/precursor complexes; (4) a modeling exercise based on the ERAP1 x-ray structure suggesting that six peptide residues would have to extend out of the MHC I groove to reach the zinc active site of ERAP1^24^; and (5) a role for ERAPs in the development of MHC I peptide repertoire^13^,^14^,^15^,^16^,^17^,^18^.

## Discussion

We have provided evidence that the N-terminus of long peptides protrudes out of the HLA-B*0801 groove. First, we showed that HLA-B*0801-bound (His)_6_ALRSRYWAI and (His)_6_AAKKKYKL were efficiently captured by Ni-NTA beads. Second, we have been unable to grow crystals of HLA-B*0801 loaded with long peptides such as (RA)_n_ALRSRYWAI, but we could grow crystals of HLA-B*0801/FLRGRKYGL. This, together with the relatively low Tm values of HLA-B*0801/precursor complexes, suggest that precursor peptides bind less optimally within the groove. Third, and consistent with this, none of our long peptides could promote the *in vitro* refolding of HLA-B*0801 molecules, as it is normally expected for peptides that fit optimally within the MHC I groove.

We showed that the MS spectra obtained after incubation of HLA-B*0801-bound precursors with ERAP1/2 are consistently different from those of the corresponding free precursors. In all cases, trimming of MHC I-bound precursors generated the correct 8mer and 9mer high-affinity peptides as the final products, i.e., peptides of optimal lengths for the size of the groove, with no evidence of smaller fragments. Moreover, HLA-B*0801-bound precursors, except for HLA-B*0801/RLRPGGKKKYKL (see below), were trimmed more slowly relative to their free forms. These results are consistent with hydrolysis taking place in a more sterically hindered environment and/or trimming proceeding by a different mechanism for MHC I-bound relative to free substrates.

Importantly, we showed that trimming of disulfide-linked (RA)_3_AAKKKYCL by ERAP1/2 yielded the final 8mer peptide, and only the 8mer, in marked contrast to free (RA)_3_AAKKKYCL which produced smaller fragments. Results from this experiment provided further support to the idea that MHC I-bound precursor peptides are true substrates of the ERAP enzymes.

The x-ray structures of ERAP1 and ERAP2 implicated that free, but not MHC I-bound, precursors are substrates of the ERAP enzymes, and also suggested that domain movements are required to generate their active conformations^22^,^23^,^24^,^25^,^26^. We suggest that conformations different from those described in these studies allow ERAPs to accommodate MHC I-bound precursors. The surprising fact that trimming of both RLRPGGKKKYKL and RPGGKKKYKL to the final GGKKKYKL 8mer (Fig. 6) occurred more slowly for the free form relative to HLA-B*0801-bound form, provide indirect support for this hypothesis. The trimming of free RLRPGGKKKYKL and RPGGKKKYKL was expected to be inefficient because of the inhibitory effects exerted by proline at P2^12^. The faster trimming in the HLA-B*0801-bound context was however unexpected, and could indeed reflect that ERAP1/2 adopts different active conformations depending on the nature, and possibly sequence lengths, of the substrates. Another important point relevant to this discussion, is that we have no knowledge of the conformations that N-terminally extended peptides adopt within the MHC I groove, and as “seen” by ERAPs. Based on five points listed in Results, we suggest that precursor peptides can bind within the groove using only a few C-terminal residues, and that the remaining N-terminal residues protrude out of the groove as modeled in Fig. 7. For a 14mer precursor (RA)_3_AAKKKYKL, this would position ~11 N-terminal residues (6 extension residues (RA)_3_ and ~5 peptide residues P1–P5) into the solvent and free to interconvert between many possible conformations. In such a model, the extension residues (RA)_3_ can be trimmed by ERAPs, with the ~5 peptide residues P1–P5 providing the necessary length for the extension residues, down to the last alanine extension (total ~6 residues, last alanine extension +P1–P5), to reach the zinc active site of ERAP (see also below). Therefore, our data are not necessarily in disagreement with the structures of ERAP1 and ERAP2 because the N-terminus extensions of a bound precursor, as depicted in our model, appears “free” to ERAPs.

Our results also showed that the stepwise trimming of HLA-B*0801/precursor complexes by ERAP1/2 consistently generated more stable complexes. These results provide a basis to explain mechanistically how ERAP1/2 can influence the formation of peptide repertoires *in vivo*. We suggest that the stepwise removal of N-terminal extension residues from MHC I-bound candidate precursors by ERAPs (see above) is accompanied by conformational changes in the groove from open conformations (for bound candidate precursors) to a closed conformation (for the final trimmed peptides). Only when precursors have been trimmed to their optimal lengths of 8–10mers, and only if the resulting final peptides can establish energetically stabilizing interactions with MHC I residues within the groove, will peptide-induced closure of the groove ensue. In this molecular cross-talk, ERAP1/2 is critical for generating *in situ* the final P1 residue needed to close the groove and, consequently, generates a stable MHC I/peptide complex. We suggest that through repeated trimming cycles “on MHC I”, the ERAP enzymes can exert an editing quality control function towards candidate precursors, by eliminating peptides (intermediates and/or finals) that cannot stabilize MHC I.

In our *in vitro* system, ERAP1/2 trims MHC I-bound precursors in the absence of the PLC. It is conceivable however that ERAPs can be accommodated within the PLC, especially because PLC proteins are presumed to bind at the C-terminal end of the MHC I groove^39^ (see Fig. 7). Independently of whether or not ERAP1/2 is part of the PLC, its editing effects on MHC I-bound candidate precursors could complement those of tapasin, as well as the more recently discovered tapasin-related protein TAPBPR^40^. We showed previously that tapasin edits peptides by acting as an energy barrier at the C-terminus of nascent MHC I molecules^4^. Here, we suggest that ERAP1/2 acts as a peptide editor by trimming MHC I-bound precursors to the correct P1 residue needed to convert the groove from an open to a closed conformation. Thus, the ERAP enzymes and tapasin regulate in distinct ways the energetics of MHC I/peptide interactions at the N- and C-terminus of the groove, respectively; both act as quality control components by favoring those peptides capable of forming stable MHC I/peptide complexes.

Finally, our analysis using ERAP1/2 (inactive/active) showed that ERAP2 has poor trimming activity towards both free and HLA-B*0801-bound precursors. We showed previously that dimer formation enhances the catalytic activity of ERAP1 while reducing that of ERAP2^29^. Given that, results obtained here suggest that trimming of precursors occurred largely from ERAP1 being activated through dimer formation with ERAP2.

In summary, our results showed that ERAP1/2 can trim MHC I-bound precursors and provided molecular mechanistic insights into how these aminopeptidases can play a role as peptide editors. Given that the assembly of MHC I/peptide complexes in the ER is completed within ~2 hours after HC synthesis, it is conceivable and even likely that the kinetics of trimming in the ER are faster than what we measured in our experiments, especially if ERAP1/2 works together with PLC proteins such as tapasin and TAPBPR. Although this is the nature of reconstituted biological systems, nonetheless the relative differences in kinetics of trimming exhibited for free versus “on MHC I” substrates that we measured indicate a distinct functional role for ERAPs towards MHC I-bound precursors. Finally, our findings provide new avenues for structural characterization of the cross-talk between ERAPs and peptide substrates, as well as open new possibilities for understanding associations between ERAP polymorphisms and autoimmune diseases.

## Methods

### Assembly of peptide-deficient MHC class I molecules

Peptide-deficient HLA-B*0801 and -B*0702 molecules were assembled from the urea denaturation of peptide-filled molecules and purified as we described previously^33^.

### Assembly of peptide-filled MHC class I molecules

Peptide-filled HLA-B*0801 and -B*0702 molecules were generated by incubating a molar excess of N-terminally extended model and natural peptides with peptide-deficient molecules (2.5 mg/mL) in 20 mM Tris-HCl, pH 7.5, 150 mM NaCl on ice. After 1 hour, the resulting MHC I/precursor complexes were washed extensively in mini-spin columns (10 kDa MWCO) to remove free peptides. The controls consisted of the corresponding free peptides incubated alone. The retentate fractions of the sample and control mixtures were characterized by MALDI-TOF MS (Voyager-DE Pro, Applied Biosystems Inc., Waltham, MA) and ESI LC MS (Shimadzu, Addison, IL) at the UIC Research Resources Center. The concentrations of MHC I/precursor complexes generated in this manner were determined and complexes used in ERAP1/2 trimming experiments. Peptide-filled MHC I molecules used to assemble peptide-deficient molecules (see above) were generated by diluting urea-solubilized inclusion bodies of class I HC (1 μM) and β_2_m (2 μM) in the presence of a synthetic peptide (10 μM) in an oxidative refolding buffer.

### Disulfide-linked HLA-B*0801E76C/(RA)_3_AAKKKYCL

We examined the crystal structure of HLA-B*0801/GRKKKYKL (PDB code 1AGB) to identify residues in HLA-B*0801 HC that are geometrically well positioned to form a disulfide bond linkage with a peptide side chain, upon mutations with cysteine residues; we selected Glu76 in the HC α1-helix and peptide residue P7. The cDNA encoding the ER-lumenal domain of HLA-B*0801E76C HC was generated by PCR using the plasmid of HLA-B*0801 HC as template (a gift of Dr. Y. Jones, University of Oxford, Oxford, UK) and QuickChange (Stratagene, La Jolla, CA). A plasmid harboring the correct DNA sequence for HLA-B*0801E76C HC was transformed into competent BL21(DE3)pLysS cells. The expression of HC mutant and β_2_m was done in *Escherichia coli*; the proteins were isolated from the cell pellets as inclusion bodies, that were then washed and solubilized in urea. Peptide-filled and peptide-deficient HLA-B*0801E76C molecules were prepared as described above.

Disulfide-linked HLA-B*0801E76C/(RA)_3_AAKKKYCL was generated in a manner similar to disulfide-linked HLA-DR1/peptide complexes^41^. First, the (RA)_3_AAKKKYCL peptide (5 μg) was incubated in 750 μM reduced glutathione, 20 mM Tris-HCl, pH 8.0, 150 mM NaCl, at 4 °C for 2 hours. Peptide-deficient HLA-B*0801E76C (12.5 μg) were then added to the reaction mixture, maintaining 750 μM reduced glutathione, followed by an additional 1 hour incubation at 4 °C. To promote disulfide bond formation, the reaction mixture was supplemented with oxidized glutathione (to 1200 μM) and incubated at 4 °C for 24 hours. The resulting disulfide-linked HLA-B*0801E76C/(RA)_3_AAKKKYCL complex was washed extensively in a mini-spin column (10 kDa MWCO) to remove free (RA)_3_AAKKKYCL. The disulfide linkage of (RA)_3_AAKKKYCL was confirmed from an analysis of HLA-B*0801E76C/(RA)_3_AAKKKYCL by MS (see Results).

The stability of HLA-B*0801E76C/(RA)_3_AAKKKYCL was tested by incubating the complex alone, in the absence of ERAP1/2, in our trimming assay buffer, i.e., 50 mM Tris-HCl, pH 7.6, 150 mM NaCl, 100 μM ZnCl_2,_ supplemented with 1 mM dithiotreitol, at 37 °C. After 10 hours, the sample was spun-down in a mini-spin column (10 kDa MWCO) and the recovered flow-through was concentrated and analyzed by MS for the presence of (RA)_3_AAKKKYCL.

### Synthetic peptides

Peptides were synthesized by solid-phase methodology on a Symphony peptide synthesizer (Protein Technologies Inc., Tucson, AZ) and purified by reverse phase high-performance liquid chromatography at the UIC Research Resources Center. The purified peptides were characterized by MALDI-TOF MS. The purified peptides were dried under vacuum and stock solutions in DMSO (10 mg/mL) were stored at −80 °C.

### Precursor peptides protrude out of the MHC I groove

HLA-B*0801/(His)_6_ALRSRYWAI and HLA-B*0801/(His)_6_AAKKKYKL (10 μg) were mixed with 10 μL of Ni-NTA slurry in 50 mM Tris-HCl, pH 7.5, 150 mM NaCl (total volume 300 μL). The mixtures were rocked at 4 °C for 2 hours, pelleted, and the beads were washed extensively with 50 mM Tris-HCl, pH 7.5, 150 mM NaCl. The beads were then boiled in SDS-PAGE loading buffer, pelleted, and the supernatants were loaded on the gel (15%). The negative controls HLA-B*0801/ALRSRYWAI and HLA-B*0801/AAKKKYKL and the positive control Ad4 E3-19 K(His)_6_ (expressed in insect cells) were treated similarly.

### ERAP beads

Human ERAP1-Jun and ERAP2-Fos cloned in baculovirus vectors were co-expressed in High Five insect cells^29^. The supernatants obtained 2 to 3 days after baculovirus infection were concentrated five times using Amicon Ultra centrifuge devices with a 50 kDa MWCO (Millipore, Molsheim, France). The concentrated supernatants were pre-cleared by incubation at 4 °C with glycine-Sepharose beads for 1 hour in the presence of protease inhibitors (cOmplete EDTA-free, Roche Diagnostics, Meylan, France). The precleared supernatant was then incubated at 4 °C with anti-ERAP2 mAb (clone 3F5) immobilized on Sepharose beads for 2 hours^42^. The ERAP1/2 beads were washed three times in 50 mM Tris-HCl, pH 7.5, 150 mM NaCl, and immediately resuspended in glycerol and frozen at −80 °C. The enzymatic activity of each batch of beads was tested by monitoring the hydrolysis of Leu-AMC and Arg-AMC (400 μM), using 350 nm excitation and 460 nm emission.

ERAP1/2 (inactive/active) beads were generated by inactivation of ERAP1 through the single-point mutation E354A in the active site^29^ (see Supplementary Fig. 4a,b). Finally, Sepharose beads carrying ERAP1 without and with the Jun modification (ERAP1 and ERAP1-Jun, respectively,) were generated by immobilization using an anti-ERAP1 mAb (clone 4D2) essentially as described above (see Supplementary Fig. 4c).

### ERAP1/2 trimming assay

To standardize the assay, each batch of ERAP1/2 beads, usually starting with 3 μL of slurry, was incubated with (RA)_3_ALRSRYWAI (1.25 μg) in 50 mM Tris-HCl, pH 7.6, 150 mM NaCl, 100 μM ZnCl_2,_ supplemented with 1 mM dithiotreitol, at 37 °C (total volume was 20 μL). After 1 hour 20 minutes, the mixture was quenched with formic acid (to 1%), spun-down for 3 minutes in a microcentrifuge (14,000 × *g*), and the supernatant analyzed by MALDI-TOF or electrospray MS (LCMS IT-TOF, Shimadzu Instruments, Columbia, MD). Depending on the intensity of the signal peak corresponding to (RA)_3_ALRSRYWAI and the overall extent of trimming, the amount of ERAP1/2 beads was adjusted to provide consistency in trimming free (RA)_3_ALRSRYWAI with each batch of beads.

The trimming of precursor peptides, either free or MHC I-bound, was carried out by incubating 1.25 μg peptide with 3–5 μL of calibrated ERAP1/2 beads in 50 mM Tris-HCl, pH 7.6, 150 mM NaCl, 100 μM ZnCl_2_, supplemented with 1 mM dithiotreitol, at 37 °C (total volume was 20 μL). At various times, the assay mixtures were spun-down for 3 minutes in a microcentrifuge (14,000 × *g*) and aliquots (5 μL) were taken from the supernatants followed by quenching with formic acid (to 1%). The aliquots were kept frozen until MS analyses. In some experiments with MHC I-bound precursors, additional ERAP1/2 beads (3–5 μL) were added to the reaction mixtures after each aliquot (see Legends). Parallel experiments were carried out for the corresponding free and MHC I-bound peptides. The trimming assay was repeated 2 to 4 times using different batches of calibrated ERAP1/2 beads.

### Stabilities of MHC I/precursor complexes and testing precursor dissociation

HLA-B*0801/ALRSRYWAI and HLA-B*0801/(RA)_3_ALRSRYWAI complexes (1.25 μg) were incubated for 10 hours at 37 °C with equal amounts of ERAP1/2 beads in 50 mM Tris-HCl, pH 7.6, 150 mM NaCl, 100 μM ZnCl_2_, supplemented with 1 mM dithiotreitol (total volume was 20 μL). The mixtures were spun-down for 3 minutes in a microcentrifuge (14,000 ×g), after which the supernatants were quenched with formic acid (to 1%) and loaded quantitatively on the native PAGE gel (12%). Coomassie-stained bands corresponding to complexes, before and after incubation, were quantified with an Odyssey FC imaging system (LI-COR Biosciences, Lincoln, NE), and signals were used to calculate percent yields of recovery.

In a modified version of this experiment, HLA-B*0801/(RA)_2_ALRSRYWAI (1.25 μg) was incubated with a molar excess of free AAKKKYKL, in the absence of ERAP1/2 beads, in 50 mM Tris-HCl, pH 7.6, 150 mM NaCl, 100 μM ZnCl_2_, supplemented with 1 mM dithiotreitol (total volume was 60 μL) at 37 °C. After 10 hours, the mixture was washed extensively in a mini-spin column (10 kDa MWCO) to remove free peptides, and the retentate fraction, containing HLA-B*0801/peptide complexes, was analyzed by MS.

### Thermolysin digests

The thermolysin digests of MHC I/peptide complexes were carried out at different temperatures for 15 minutes at enzyme:substrate ratio of 1:300 (W/W) in 20 mM Tris-HCl, pH 7.5, 150 mM NaCl, 2 mM CaCl_2_, and 15% glycerol. The reaction mixtures were quenched with EDTA (to 10 mM) and cooled on ice followed by analysis on SDS-PAGE gel (15%).

### Circular dichroism

Thermal denaturation curves of purified HLA-B*0801/peptide complexes, generated from two independent experiments, were obtained in duplicate on a Jasco J-715 spectropolarimeter (Jasco Inc., Rochester, NY) equipped with an external water bath. Curves were obtained by monitoring the change in signal at 218 nm in the range 25° to 85 °C using a scan rate of 1 °C per minute. A 1 cm water-jacketed quartz glass cuvette was used for all measurements. The protein concentrations were 0.18 mg/mL in 20 mM Tris-HCl, pH 7.5, 150 mM NaCl. Ellipticities are expressed on a molar residue basis. The thermal denaturation temperatures, Tm values, were determined from the first-derivative of the denaturation curves.

## Additional Information

**How to cite this article**: Li, L. *et al.* ERAP1-ERAP2 dimers trim MHC I-bound precursor peptides; implications for understanding peptide editing. *Sci. Rep.* **6**, 28902; doi: 10.1038/srep28902 (2016).

## Supplementary Material

Supplementary Information

## References

[b1] Hulpke S. & Tampe R. The MHC I loading complex: a multitasking machinery in adaptive immunity. Trends Biochem. Sci. 38, 412–420 (2013). 23849087 10.1016/j.tibs.2013.06.003

[b2] Weimershaus M., Evnouchidou I., Saveanu L. & van Endert P. Peptidase trimming MHC class I ligands. Curr. Opin. Immunol. 25, 90–96 (2013). 23089230 10.1016/j.coi.2012.10.001

[b3] Howarth M., Williams A., Tolstrup A. B. & Elliott T. Tapasin enhances MHC class I peptide presentation according to peptide half-life. Proc. Natl. Acad. Sci. USA 101, 11737–11742 (2004). 15286279 10.1073/pnas.0306294101 PMC511045

[b4] Chen M. & Bouvier M. Analysis of interactions in a tapasin/class I complex provides a mechanism for peptide selection. EMBO J. 26, 1681–1690 (2007). 17332746 10.1038/sj.emboj.7601624 PMC1829385

[b5] Sieker F., Springer S. & Zacharias M. Comparative molecular dynamics analysis of tapasin-dependent and -independent MHC class I alleles. Protein Sci. 16, 299–308. (2007). 17242432 10.1110/ps.062568407 PMC2203297

[b6] Wearsch P. A. & Cresswell P. Selective loading of high-affinity peptides onto major histocompatibility complex class I molecules by the tapasin-ERp57 heterodimer. Nat. Immunol. 8, 873–881 (2007). 17603487 10.1038/ni1485

[b7] Praveen P. V., Yaneva R., Kalbacher H. & Springer S. Tapasin edits peptides on MHC class I molecules by accelerating peptide exchange. Eur. J. Immunol. 40, 214–224 (2010). 20017190 10.1002/eji.200939342

[b8] Rizvi S. M. & Raghavan M. Mechanism of function of tapasin, a critical major histocompatibility complex class I assembly factor. Traffic 11, 332–347 (2010). 20070606 10.1111/j.1600-0854.2009.01025.x PMC2983092

[b9] van Endert P. Post-proteasomal and proteasome-independent generation of MHC class I ligands. Cell Mol. Life Sci. 68, 1553–1567 (2011). 21390545 10.1007/s00018-011-0662-1 PMC11115176

[b10] Saric T. *et al.* An IFN-γ-induced aminopeptidase in the ER, ERAP1, trims precursors to MHC class I-presented peptides. Nat. Immunol. 3, 1169–1176 (2002). 12436109 10.1038/ni859

[b11] Serwold T., Gonzalez F., Kim J., Jacob R. & Shastri N. ERAAP customizes peptides for MHC class I molecules in the endoplasmic reticulum. Nature 419, 480–483 (2002). 12368856 10.1038/nature01074

[b12] York I. A. *et al.* The ER aminopeptidase ERAP1 enhances or limits antigen presentation by trimming epitopes to 8–9 residues. Nat. Immunol. 3, 1177–1184 (2002). 12436110 10.1038/ni860

[b13] Hammer G. E., Gonzalez F., Champsaur M., Cado D. & Shastri N. The aminopeptidase ERAAP shapes the peptide repertoire displayed by major histocompatibility complex class I molecules. Nat. Immunol. 7, 103–112 (2006). 16299505 10.1038/ni1286

[b14] Yan J. *et al.* *In vivo* role of ER-associated peptidase activity in tailoring peptides for presentation by MHC class Ia and class Ib molecules. J. Exp. Med. 203, 647–659 (2006). 16505142 10.1084/jem.20052271 PMC2118255

[b15] Hammer G. E., Gonzalez F., James E., Nolla H. & Shastri N. In the absence of aminopeptidase ERAAP, MHC class I molecules present many unstable and highly immunogenic peptides. Nat. Immunol. 8, 101–108. (2007). 17128277 10.1038/ni1409

[b16] Firat E. *et al.* The role of endoplasmic reticulum-associated aminopeptidase 1 in immunity to infection and in cross-presentation. J. Immunol. 178, 2241–2248 (2007). 17277129 10.4049/jimmunol.178.4.2241

[b17] Blanchard N. *et al.* Endoplasmic reticulum aminopeptidase associated with antigen processing defines the composition and structure of MHC class I peptide repertoire in normal and virus-infected cells. J. Immunol. 184, 3033–3042 (2010). 20173027 10.4049/jimmunol.0903712 PMC3087292

[b18] Kanaseki T. *et al.* ERAPP and tapasin independently edit the amino and carboxyl termini of MHC class I peptides. J. Immunol. 191, 1547–1555 (2013). 23863903 10.4049/jimmunol.1301043 PMC3735839

[b19] Kanaseki T., Blanchard N., Hammer G. E., Gonzalez F. & Shastri N. ERAAP synergizes with MHC class I molecules to make the final cut in the antigenic peptide precursors in the endoplasmic reticulum. Immunity 25, 795–806 (2006). 17088086 10.1016/j.immuni.2006.09.012 PMC2746443

[b20] Falk K., Rotzschke O. & Rammensee H.-G. Cellular peptide composition governed by major histocompatibility complex class I molecules. Nature 348, 248–251 (1990). 2234092 10.1038/348248a0

[b21] Chang A. C., Momburg F., Bhutani N. & Goldberg A. L. The ER aminopeptidase, ERAP1, trims precursors to lengths of MHC class I peptides by a “molecular ruler” mechanism. Proc. Natl. Acad. Sci. USA 102, 17107–17112 (2005). 16286653 10.1073/pnas.0500721102 PMC1287962

[b22] Gandhi A., Lakshminarasimhan D., Sun Y. & Guo H.-C. Structural insights into the molecular ruler mechanism of the endoplasmic reticulum aminopeptidase ERAP1. Sc. Reports 186, 1–6 (2011). 10.1038/srep00186 PMC3240994 22355701

[b23] Kochan G. *et al.* Crystal structures of the endoplasmic reticulum aminopeptidase-1 (ERAP1) reveal the molecular basis for N-terminal peptide trimming. Proc. Natl. Acad. Sc. USA 108, 7745–7750 (2011). 21508329 10.1073/pnas.1101262108 PMC3093473

[b24] Nguyen T. T. *et al.* Structural basis for antigenic peptide precursor processing by the endoplasmic reticulum aminopeptidase ERAP1. Nat. Struct. Molec. Biol. 18, 604–613 (2011). 21478864 10.1038/nsmb.2021 PMC3087843

[b25] Birtley J. R., Saridakis E., Stratikos E. & Mavridis I. M. The crystal structure of human endoplasmic reticulum aminopeptidase 2 reveals the atomic basis for distinct roles in antigen processing. Biochem. 51, 286–295 (2012). 22106953 10.1021/bi201230p

[b26] Mpakali A. *et al.* Structural basis for antigenic peptide recognition and processing by endoplasmic reticulum (ER) aminopeptidase 2. J. Biol. Chem. 290, 26021–26032 (2015). 26381406 10.1074/jbc.M115.685909 PMC4646255

[b27] Reeves E., Edwards C. J., Elliott T. & James E. Naturally occurring *ERAP1* haplotypes encode functionally distinct alleles with fine substrate specificity. J. Immunol. 191, 35–43 (2013). 23733883 10.4049/jimmunol.1300598 PMC3785127

[b28] Alvarez-Navarro C. & Lopez de Castro J. A. ERAP1 structure, function, and pathogenetic role in ankylosing spondylitis and other MHC-associated diseases. Molec. Immunol. 57, 12–21 (2014). 23916068 10.1016/j.molimm.2013.06.012

[b29] Evnouchidou I., Weimershaus M., Saveanu L. & van Endert P. ERAP1-ERAP2 dimerization increases peptide-trimming efficiency. J. Immunol. 193, 901–908 (2014). 24928998 10.4049/jimmunol.1302855

[b30] Suhrbier A., Schmidt C. & Fernan A. Prediction of an HLA-B8-restricted influenza epitope by motif. Immunol. 79, 171–173 (1993). PMC1422045 7685314

[b31] Schatz M. M. *et al.* Characterizing the N-terminal processing motif of MHC class I ligands. J. Immunol. 180, 3210–3217 (2008). 18292545 10.4049/jimmunol.180.5.3210

[b32] Phillips R. E. *et al.* Human immunodeficiency virus genetic variation that can escape cytotoxic T cell recognition. Nature 354, 453–459 (1991). 1721107 10.1038/354453a0

[b33] Bouvier M. & Wiley D. C. Structural characterization of a soluble and partially folded class I major histocompatibility heavy chain/β_2_m heterodimer. Nat. Struc. Biol. 5, 377–384 (1998). 10.1038/nsb0598-377 9587000

[b34] van Endert P. M. *et al.* The peptide-binding motif for the human transporter associated with antigen processing. J. Exp. Med. 182, 1883–1895 (1995). 7500034 10.1084/jem.182.6.1883 PMC2192244

[b35] Jin X. *et al.* Identification of subdomainant cytotoxic T lymphocyte epitopes encoded by autologous HIV type I sequence, using dendritic cell stimulation and computer-drive algorithm. AIDS Res. Hum. Retrov. 16, 67–76 (2000). 10.1089/088922200309610 10628818

[b36] Bouvier M. & Wiley D. C. Importance of peptide amino and carboxyl termini to the stability of MHC class I molecules. Science 265, 398–402 (1994). 8023162 10.1126/science.8023162

[b37] Garstka M. A. *et al.* The first step of peptide selection in antigen presentation by MHC class I molecules. Proc. Natl. Acad. Sc. USA 112, 1505–1510 (2015). 25605945 10.1073/pnas.1416543112 PMC4321303

[b38] Glithero A. *et al.* The crystal structure of H-2D^b^ complexed with a partial peptide epitope suggests a major histocompatibility complex class I assembly intermediate. J. Biol. Chem. 281, 12699–12704 (2006). 16478731 10.1074/jbc.M511683200

[b39] Bouvier M. Accessory proteins and the assembly of human class I MHC molecules: a molecular and structural perspective. Molec. Immunol. 39, 697–706 (2003). 12531281 10.1016/s0161-5890(02)00261-4

[b40] Hermann C. *et al.* TAPBPR alters MHC class I peptide presentation by functioning as a peptide exchange catalyst. eLife 09617 (2015). 10.7554/eLife.09617 PMC4718805 26439010

[b41] Anders A.-K. *et al.* HLA-DM captures partially empty HLA-DR molecules for catalyzed removal of peptide. Nat. Immunol. 12, 54–61 (2011). 21131964 10.1038/ni.1967 PMC3018327

[b42] Saveanu L. *et al.* Concerted peptide trimming by human ERAP1 and ERAP2 aminopeptidase complexes in the endoplasmic reticulum. Nat. Immunol. 6, 689–697 (2005). 15908954 10.1038/ni1208

[b43] Pettersen E. F. *et al.* UCSF Chimera - a visualization system for exploratory research and analysis. J. Comput. Chem. 25, 1605–1612 (2004). 15264254 10.1002/jcc.20084

