# Supplementary material for: ERAP1-ERAP2 dimers trim MHC I-bound precursor peptides; implications for understanding peptide editing
Source: Sci Rep. 2016 Aug 12;6:28902. doi: 10.1038/srep28902 (PMC4981824; doi:10.1038/srep28902)
Supplement: Supplementary Information [file srep28902-s1.pdf]

# ERAP1-ERAP2 dimers trim MHC I-bound precursor peptides; implications for understanding peptide editing

Lenong Li[1,3], Hanna Chen[1,3], Mirjana Weimershaus[2], Irini Evnouchidou[2], Peter van Endert[2], and Marlene Bouvier[1,*]

**Supplementary Figure 1, Related to Figure 2** N-terminal trimming of free and HLA-B*0801-bound $(RA)_2$ALRSRYWAI by ERAP1/2. (**a**) Free $(RA)_2$ALRSRYWAI 13mer was incubated with ERAP1/2 at 37°C. An aliquot was taken from the mixture after 1 hour 20 minutes. (**b**) HLA-B*0801-bound $(RA)_2$ALRSRYWAI 13mer was incubated with ERAP1/2 at 37°C. Aliquots were taken from the mixture after 2, 6, and 10 hours. Initially, the enzyme:substrate ratio was as identical as possible in (**a**) and (**b**), but additional ERAP1/2 was added in (**b**) after 2 and 6 hours. (**c**) HLA-B*0801-bound $(RA)_2$ALRSRYWAI 13mer was incubated alone for 10 hours as in (**b**), in the absence of ERAP1/2. (**d**) HLA-B*0801-bound $(RA)_2$ALRSRYWAI 13mer was incubated with an excess of free AAKKKYKL 8mer ($m/z$ = 949) for 10 hours as in (**b**), in the absence of ERAP1/2. The starting precursor peptide and its fragments are identified by their $m/z$ values.

**Supplementary Figure 2, Related to Figure 2** N-terminal trimming of free and HLA-B*0801-bound $(RA)_3$AAKKKYKL by ERAP1/2. (**a**) Free $(RA)_3$AAKKKYKL 14mer was incubated with ERAP1/2 at 37°C. An aliquot was taken from the mixture after 1 hour 20 minutes. (**b**) HLA-B*0801-bound $(RA)_3$AAKKKYKL 14mer was incubated with ERAP1/2 at 37°C. Aliquots were taken from the mixture after 2, 6, and 10 hours. Initially, the enzyme:substrate ratio was as identical as possible in (**a**) and (**b**), but additional ERAP1/2 was added in (**b**) after 2 and 6 hours. The starting precursor peptide and its fragments are identified by their $m/z$ values.

**Supplementary Figure 3, Related to Figure 2** N-terminal trimming of free and HLA-B*0801-bound $(RA)_2$AAKKKYKL by ERAP1/2. (**a**) Free $(RA)_2$AAKKKYKL 12mer was incubated with ERAP1/2 beads at 37°C. An aliquot was taken from the mixture after 1 hour 20 minutes. (**b**) HLA-B*0801-bound $(RA)_2$AAKKKYKL 12mer was incubated with ERAP1/2 at 37°C. Aliquots were taken from the mixture after 2, 6, and 10 hours. Initially, the enzyme:substrate ratio was as identical as possible in (**a**) and (**b**), but additional ERAP1/2 were added in (**b**) after 2 and 6 hours. The starting precursor peptide and its fragments are identified by their *m/z* values.

**Supplementary Figure 4, related to Methods** N-terminal trimming of free and HLA-B*0801-bound $(RA)_3$ALRSRYWAI by ERAP1/2 carrying inactive ERAP1. (**a**) Free $(RA)_3$ALRSRYWAI 15mer was incubated with ERAP1/2 (inactive/active) at 37°C. An aliquot was taken from the mixture after 1 hour 20 minutes. (**b**) HLA-B*0801-bound $(RA)_3$ALRSRYWAI 15mer was incubated with ERAP1/2 (inactive/active) at 37°C. Aliquots were taken after 2 and 10 hours. Initially, the enzyme:substrate ratio was as identical as possible in (**a**) and (**b**), but additional ERAP1/2 (inactive/active) was added in (**b**) after 2 and 6 hours. (**c**) Free $(RA)_3$ALRSRYWAI 15mer was incubated with ERAP1 (without Jun) and ERAP1-Jun at 37°C. An aliquot was taken from the mixture after 1 hour 20 minutes. The starting precursor peptides and their fragments are identified by their *m/z* values.

**Supplementary Figure 5, Related to Figure 4** N-terminal trimming of HLA-B*0801-bound LAKLRNKLVI 10mer and AKLRNKLVI 9mer by ERAP1/2. (**a**) HLA-

B*0801/LAKLRNKLVI was incubated with ERAP1/2 at 37°C. Aliquots were taken from the mixture after 2 and 4 hours. (**b**) Same experiment as in (**a**) for HLA-B*0801/AKLRNKLVI. The enzyme:substrate ratio was as identical as possible in (**a**) and (**b**), and additional ERAP1/2 was added in (**a**) and (**b**) after 2 and 4 hours. The starting precursor peptides and its fragments are identified by their *m/z* values.

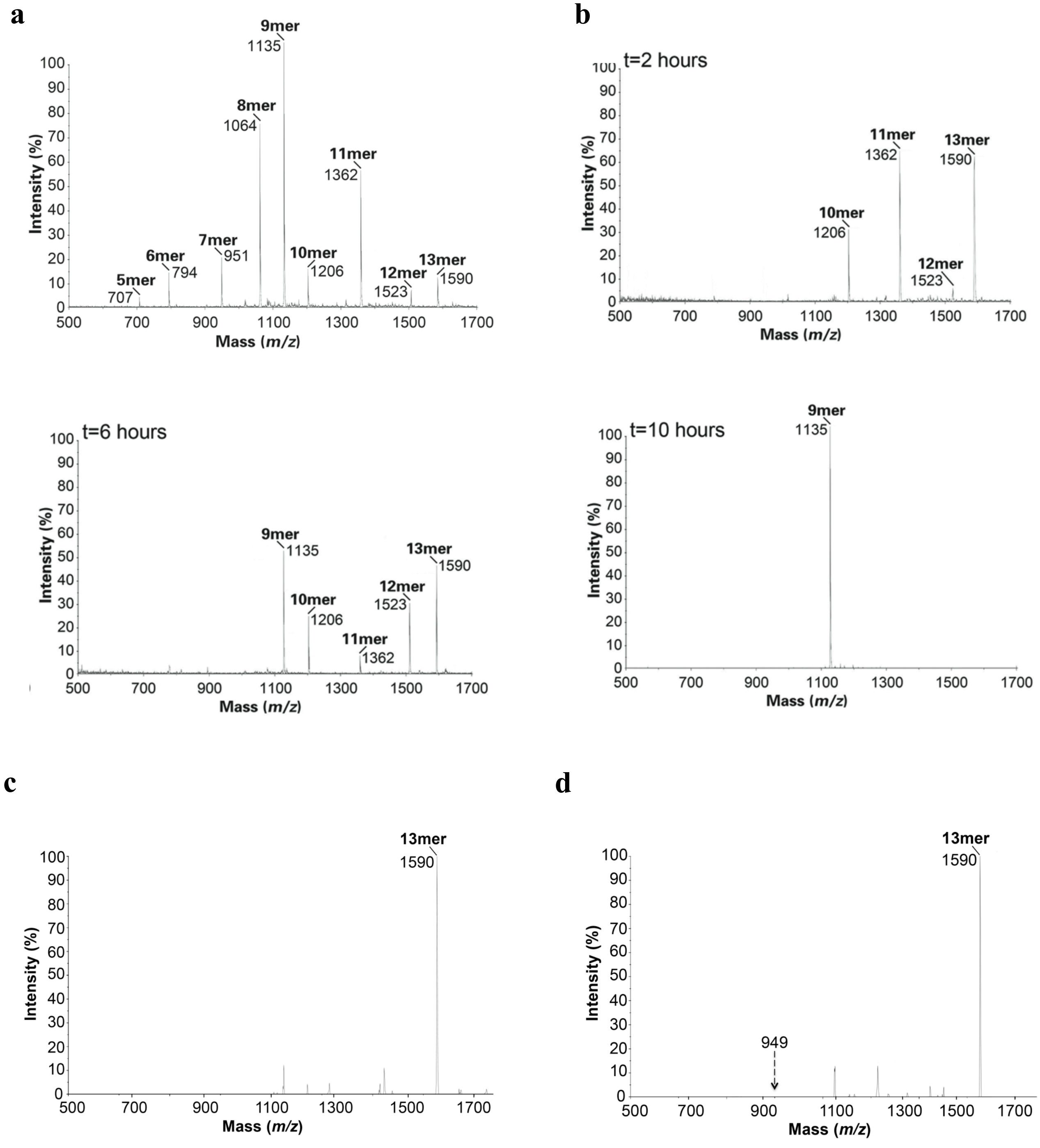


Supplementary Figure 1

a

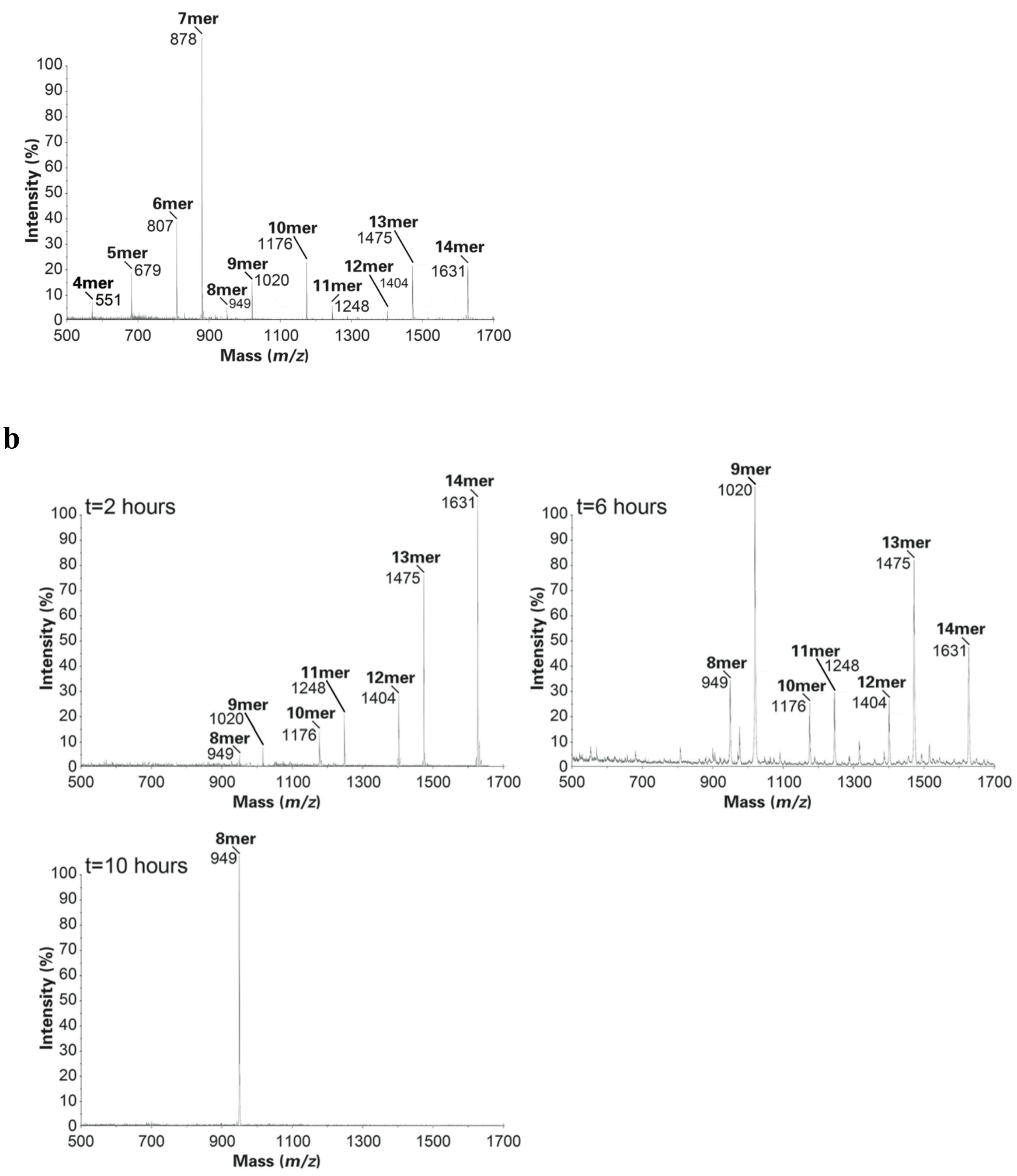


Supplementary Figure 2

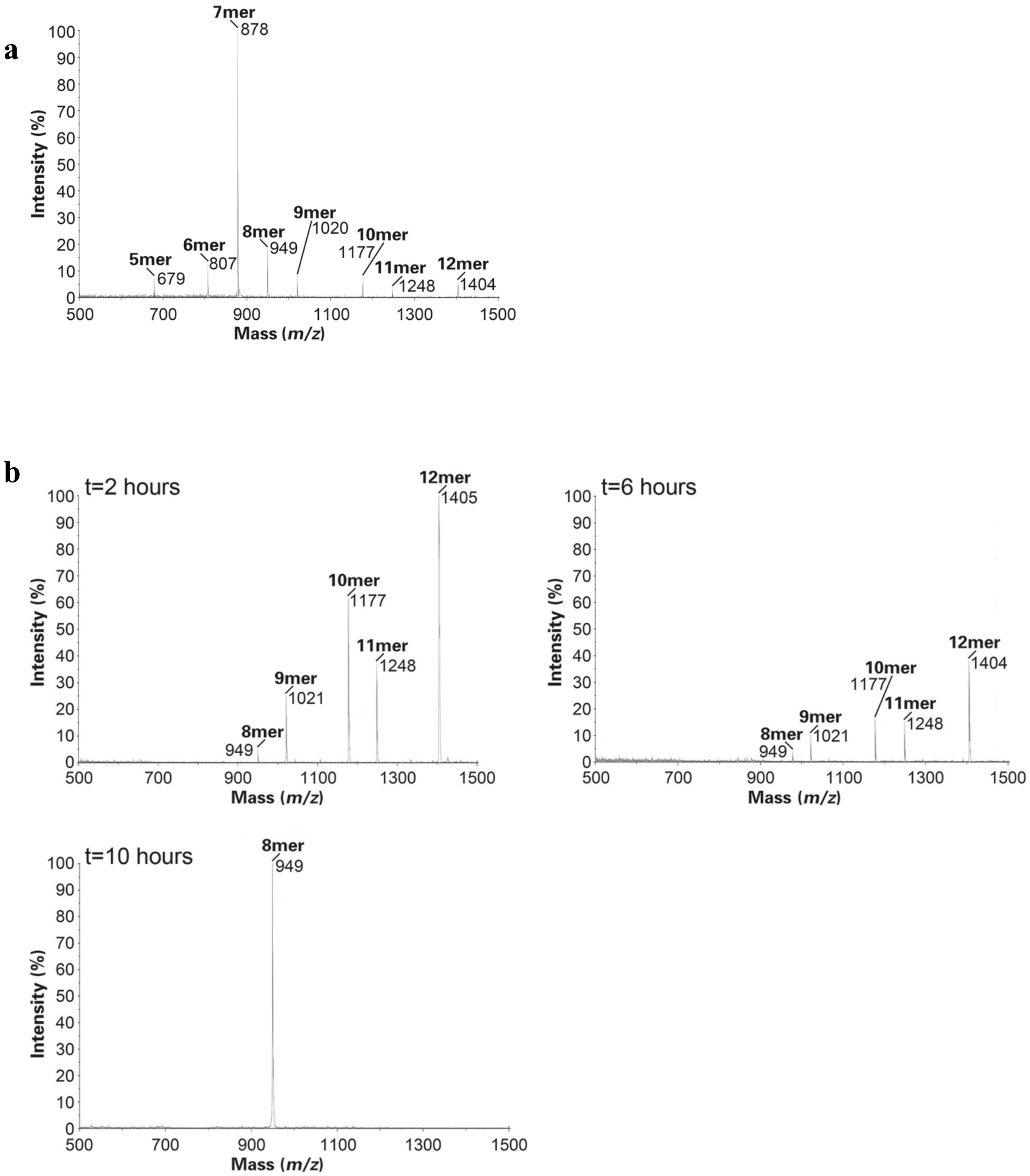


Supplementary Figure 3

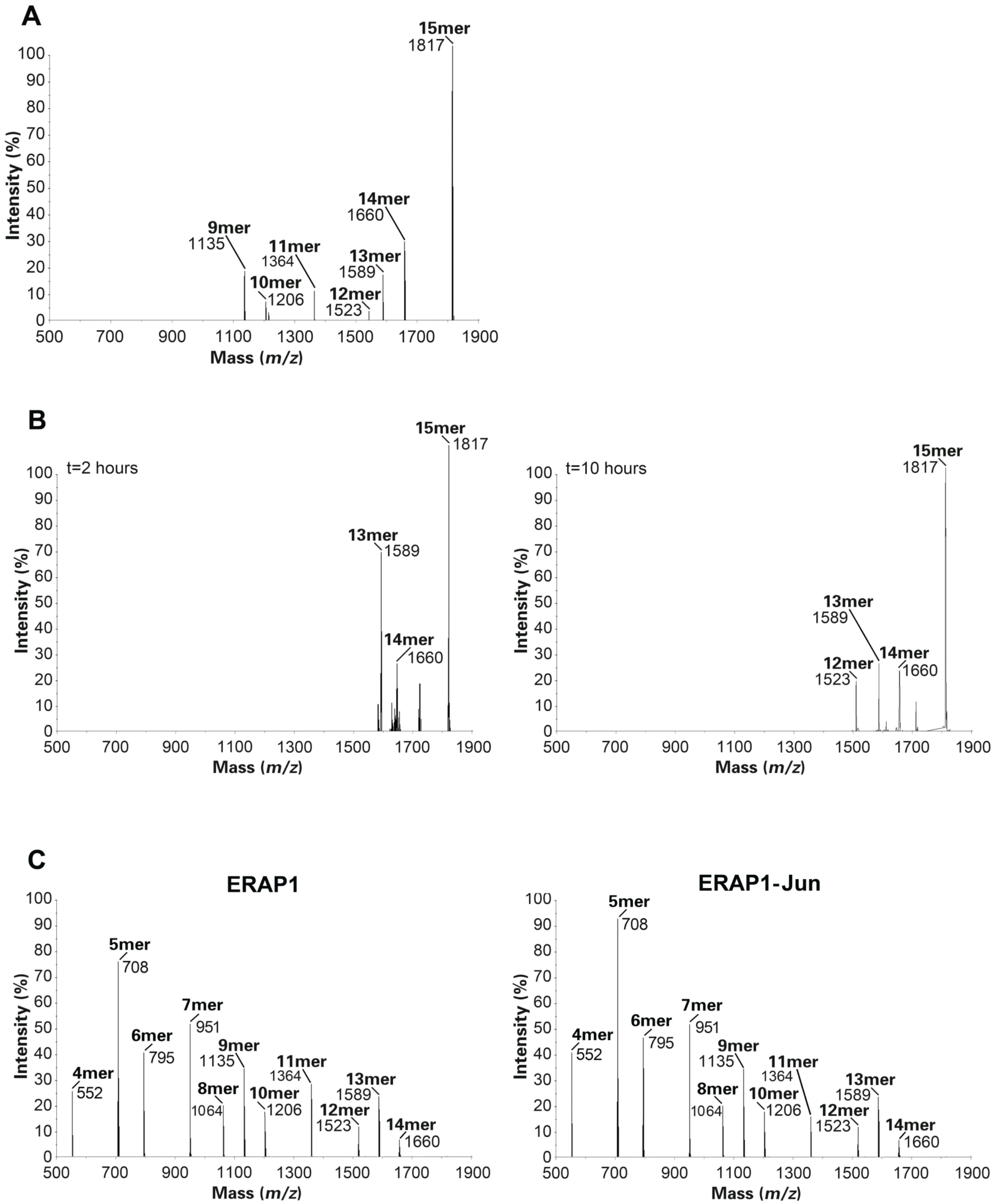


Supplementary Figure 4

**a**

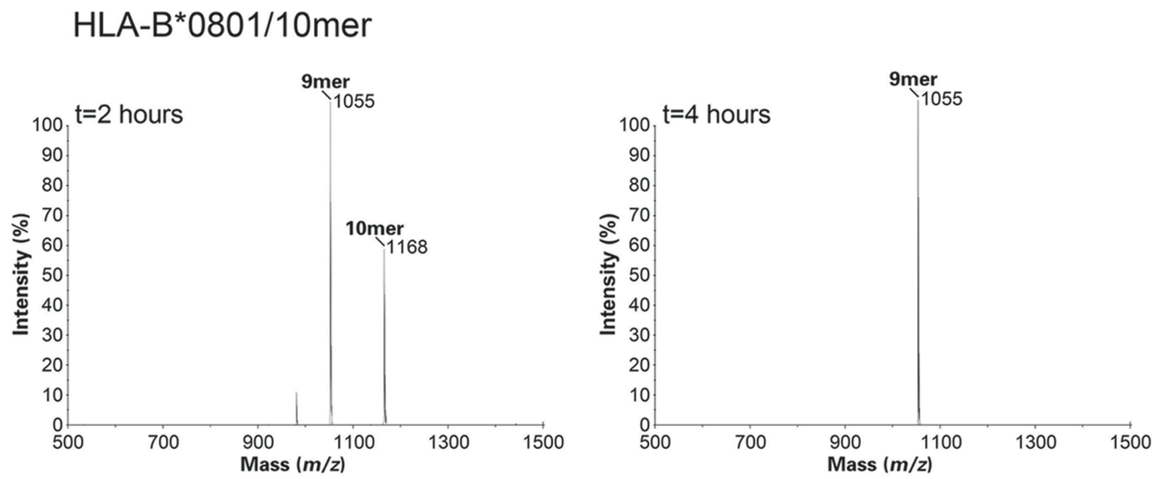


**b**

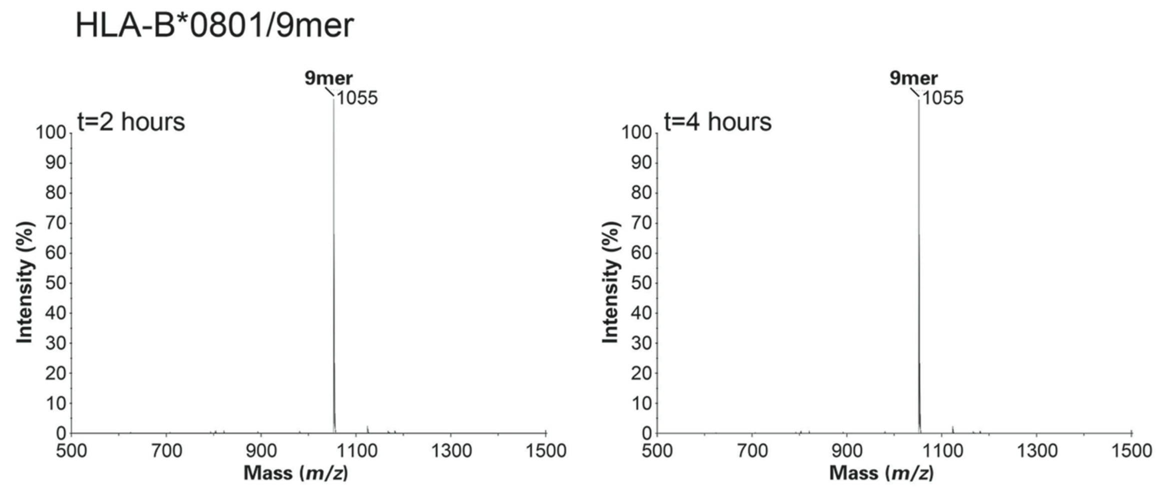


Supplementary Figure 5
